# Atomically thin noble metal dichalcogenide: a broadband mid-infrared semiconductor

**DOI:** 10.1038/s41467-018-03935-0

**Published:** 2018-04-18

**Authors:** Xuechao Yu, Peng Yu, Di Wu, Bahadur Singh, Qingsheng Zeng, Hsin Lin, Wu Zhou, Junhao Lin, Kazu Suenaga, Zheng Liu, Qi Jie Wang

**Affiliations:** 1 Centre for OptoElectronics and Biophotonics, School of Electrical and Electronic Engineering & The Photonics Institute, Nanyang Technological University, 50 Nanyang Avenue, Singapore, 639798 Singapore; 2 Centre for Programmable Materials, School of Materials Science and Engineering, Nanyang Technological University, 50 Nanyang Avenue, Singapore, 637371 Singapore; 3 Centre for Advanced 2D Materials and Graphene Research Centre, National University of Singapore, Singapore, 117546 Singapore; 4 Department of Physics, National University of Singapore, Singapore, 117542 Singapore; 5 School of Physics Science, CAS Key Laboratory of Vacuum Physics, University of Chinese Academy of Sciences, 100049 Beijing, China; 6 National Institute of Advanced Industrial Science and Technology (AIST), Tsukuba, 305-8565 Japan; 7 NOVITAS, Nanoelectronics Centre of Excellence, School of Electrical and Electronic Engineering, Nanyang Technological University, Singapore, 639798 Singapore

## Abstract

The interest in mid-infrared technologies surrounds plenty of important optoelectronic applications ranging from optical communications, biomedical imaging to night vision cameras, and so on. Although narrow bandgap semiconductors, such as Mercury Cadmium Telluride and Indium Antimonide, and quantum superlattices based on inter-subband transitions in wide bandgap semiconductors, have been employed for mid-infrared applications, it remains a daunting challenge to search for other materials that possess suitable bandgaps in this wavelength range. Here, we demonstrate experimentally for the first time that two-dimensional (2D) atomically thin PtSe_2_ has a variable bandgap in the mid-infrared via layer and defect engineering. Here, we show that bilayer PtSe_2_ combined with defects modulation possesses strong light absorption in the mid-infrared region, and we realize a mid-infrared photoconductive detector operating in a broadband mid-infrared range. Our results pave the way for atomically thin 2D noble metal dichalcogenides to be employed in high-performance mid-infrared optoelectronic devices.

## Abstract (web summary)

The mid-infrared technologies are essential to various applications but suffer from limited materials with suitable bandgap. Here the authors demonstrate that two-dimensional atomically thin PtSe_2_ with variable bandgaps in the mid-infrared via layer and defect engineering is highly promising for mid-infrared optoelectronics.

## Introduction

The enthusiasm for research on mid-infrared (mid-IR) radiation that contains fingerprints of the molecular vibrations and transparent windows in the atmosphere is driven by the tremendous applications ranging from optical communications, infrared imaging to analytical sciences^1^. Commercial Mercury Cadmium Telluride (MCT) made of CdTe and HgTe alloy, by far the paradigmatic candidate for mid-IR applications, exhibits robust performances, and the bandgap is varied by adjusting the alloy compositions, or by externally changing the operation temperatures^2^. InSb is also an extensively explored narrow bandgap semiconductor for short-wave mid-IR applications, and the operation regime can be extended to ~7 μm via nitrogen doping^3^. Apart from these materials based on interband transitions, compound semiconductor superlattices with alternate layers of different semiconductors have been developed in the past few decades for mid-IR optoelectronics, based on intersubband transitions^4,5^. The present mid-IR materials are intensively stuck with environmental toxicity, high cost, and complex fabrication processes.

The urgency for exploring alternative candidate materials in the mid-IR range has been raised with insistence. The two-dimensional (2D) material is an emerging platform with atomical thickness and exceptional properties, which has revolutionized material science, chemistry, and physics^6^. For instance, graphene, a 2D crystal with honeycomb-arranged carbon atom layer, has attracted enormous interests in the mid-IR optoelectronics due to its unique optoelectronic properties, such as broadband absorption, ultrahigh carrier mobility, etc.^7^. However, the low-absorption coefficient and short-carrier lifetime are major constraints for developing versatile mid-infrared optoelectronic devices, even though graphene nanoribbons have been employed to either open a suitable bandgap or excite graphene plasmons for enhancing the mid-infrared light−matter interactions^8–10^. The existing challenges stimulate the search for alternative 2D materials with an intrinsic narrow bandgap. Black phosphorene (BP), which is recently rediscovered from the perspective of a 2D layered material, has a layer-dependent bandgap from 0.3 to 2.0 eV. Although multilayer BP is suitable for mid-infrared photodetectors and modulators^11–13^, it only covers up to a wavelength of ~4.1 μm (*hυ* ~ 0.3 eV) and suffers from relatively poor environmental stability^12^. On the other hand, traditional 2*H*-phase transition metal dichalcogenides (TMDCs), MX_2_ (M=Mo, W; X=S, Se), are not suitable for mid-infrared photonic applications because of the wide bandgap that only covers the visible range^14^. As a result, most of the currently explored TMDCs are not appropriate for this lower-energy spectral range via interband optical transitions.

Extending 2D materials to the spectral range of mid-infrared irradiation has experienced an impetus in terms of developing novel theoretical and technological strategies to provide new insights into related phenomena^15^. Recently, the noble metal dichalcogenides (such as PtS_2_, PtSe_2_, PdS_2_, PdSe_2_, etc.) present a foray into promising candidates due to their potentially suitable bandgaps for the mid-infrared photonic and optoelectronic applications, and high environmental stability^16^. In this work, we successfully demonstrate high-performance mid-infrared materials from bilayer PtSe_2_ mechanically exfoliated from its bulk counterpart, which is synthesized by a self-designed chemical vapor transport (CVT) method. Compared with other fabrication strategies that we have also demonstrated in this work, such as chemical vapor deposition (CVD) and direct selenization of platinum substrate, our designed CVT method has the most leverage to realize high-quality, large-area, and layer-controlled PtSe_2_ atomic layers for optoelectronic devices. Intriguingly, the bandgap can also be efficiently tuned through defect engineering via growth-condition controls and/or plasma treatments, which is verified by both theoretical calculations and mid-IR optoelectronic measurements. High-performance photoconductive detectors with a remarkable photoresponse in a broadband spectrum region from the visible to the mid-infrared are achieved, using bilayer PtSe_2_ as an activating material. Our results reveal a roadmap toward future development of high-performance 2D material for photonic devices, such as mid-infrared cameras, photodetectors, modulators etc.

## Results

### PtSe2 atomic layers fabrication methods and characterizations

**Fig. 1:**
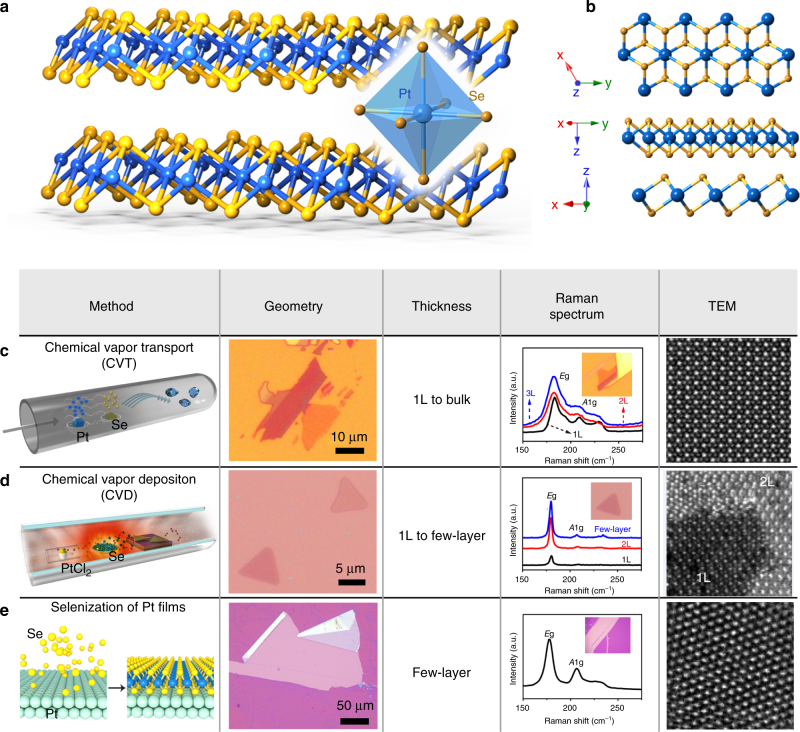
Synthesis and characterizations of PtSe_2_ by three different fabrication methods. **a** Crystal structure of PtSe_2_ and **b** its three-dimensional (3D) side views. **c** Illustrative diagram of experimental setup for the growth of PtSe_2_ single crystals by CVT method, optical microscope image, Raman spectroscopy, and HRTEM image of atomic PtSe_2_ layers via mechanical exfoliation. **d** Schematic diagram of experimental setup, optical microscope image, Raman spectroscopy, and HRTEM image of atomic PtSe_2_ layers fabricated by CVD method. **e** Growth process, optical microscope image, Raman spectroscopy, and HRTEM image of the few-layer (FL) PtSe_2_ via direct selenization of platinum substrate

PtSe_2_ crystal possesses a typical 1*T*-type hexagonal crystal structure with *P-3m1* space group, as shown in Fig. 1a, b_._ It consists of three atomic layers stacking in the order of Se-Pt-Se, which are held together by weak Van Der Waals forces. Within a single PtSe_2_ layer, Se atoms are strongly bonded with Pt atoms forming an octahedral prismatic local coordination structure, where Pt atoms lie at the center of the coordination unit. The PtSe_2_ octahedra are connected to each other by sharing Se-Se edges, as shown in Supplementary Fig. 1. The layered crystal structure of PtSe_2_, analogous to the traditional TMDCs, enables the fabrication of atomic layers by proper exfoliation strategies. However, obtaining high-quality PtSe_2_ atomic layers with designed bandgap and thickness is critical toward its practical applications. In this paper, we report a custom-designed CVT setup to synthesize PtSe_2_ single crystal, followed by a mechanical exfoliation method to fabricate the atomic layers, as shown in Fig. 1c. The source material evaporation and reaction process are controlled by adjusting the temperature zone in the vacuum tube. High-resolution transmission electron microscope (HRTEM) of PtSe_2_ atomic layers indicates the high-quality of the fabricated thin layers. For comparison, we also design two alternative methods to synthesize PtSe_2_ atomic layers: CVD and direct selenization of Pt substrate. In the CVD process, PtSe_2_ atomic layers are achieved by evaporating PtCl_2_ and selenide source materials, followed by reaction and deposition on the Si/SiO_2_ wafer. The Raman spectrum, as shown in Fig. 1d, indicates sharp characteristic peaks; however, the HRTEM image shows some large holes and structural defects. In addition, direct selenization of platinum substrate, as shown in Fig. 1e, is also proved to be an efficient strategy to synthesize high-quality PtSe_2_ thin films, in which the thickness can be tuned by the growth temperature. The Raman spectrum shows similar *E*_g_ and *A*_1g_ peaks, and the HRTEM image is found to be fully consistent with the crystal structure of 1*T*-PtSe_2_^17^.

From the above three methods and the corresponding characterizations presented here, we can state that the CVD method is feasible to fabricate PtSe_2_ atomic layers from monolayer to few layers on the conventional Si/SiO_2_ wafer, but the size and quality of the PtSe_2_ atomic layers are not suitable for optoelectronic device applications. Even though the direct selenization of Pt substrate results in large size and high-quality PtSe_2_, only thin films of PtSe_2_ on the Pt substrate can be fabricated by this method, which is also not acceptable for optoelectronic device applications. On the contrary, the CVT method followed by a flexible mechanical exfoliation process is capable of fabricating PtSe_2_ atomic layers from monolayer to few layers with high-quality. As a result, we employ the designed CVT method, as shown in Fig. 1c., to explore the mid-IR optoelectronics properties in this work.

**Fig. 2:**
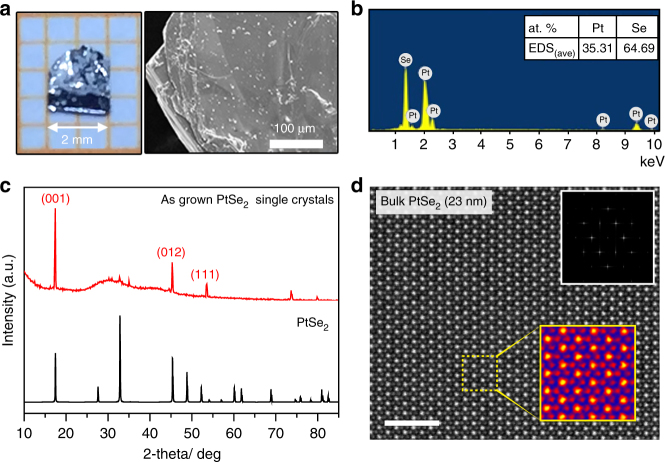
Synthesis and characterization of PtSe_2_ crystals fabricated through the CVT method. **a** Optical microscope image and scanning electron microscope (SEM) image of the synthesized PtSe_2_ flakes. **b** EDS of the PtSe_2_ flakes indicating the atomic ratio of Pt/Se. **c** X-ray diffraction spectra of the CVT grown, large PtSe_2_ crystals (up, red), and 1*T*-PtSe_2_ standard card (bottom, black). **d** Transmission electron microscopy characterization, Fast Fourier transform of the atomic resolution scanning transmission electron microscope (STEM), Z-contrast image (the upper right corner), and STEM Z-contrast image of the exfoliated PtSe_2_ few layers. The scale bar is 2 nm

The as-grown single crystals of PtSe_2,_ up to a size of 2–3 mm, is shown in Fig. 2a with a flat and wrinkle-free surface. The energy-dispersive X-ray spectroscopy (EDS) spectrum in Fig. 2b indicates the pure chemical composition of the exfoliated PtSe_2_ flakes without the residues of the catalyzers. However, the atomic ratio is smaller than that in the perfect PtSe_2_ crystal, indicating that Se is normally deficient in the crystal fabricated by the CVT method. Figure 2c shows the X-ray diffraction (XRD) pattern of the fabricated PtSe_2_ crystals. The X-ray pattern matches very well with the previous studies^17,18^, and the three major characteristic peaks are observed, while other peaks are counteracted due to the layered structure and special orientation (001) of the PtSe_2_ crystal. In addition, the strong, narrow peaks for (001), (012), and (111) demonstrate the high crystal quality of the synthesized samples. The EDS further confirms that pure phase of as-grown PtSe_2_ has been successfully obtained by the CVT method. The quality of PtSe_2_ sample is systematically characterized by TEM, as shown in Fig. 2d, to clarify its quality and atomic structure, from which we can obtain the lattice constant of ~3.8 Å, in excellent agreement with the theoretical value and previous reports^17^. The TEM, EDS, as well as the XRD pattern in Fig. 2 demonstrates the successful growth of highly crystallined PtSe_2_ flakes.

**Fig. 3:**
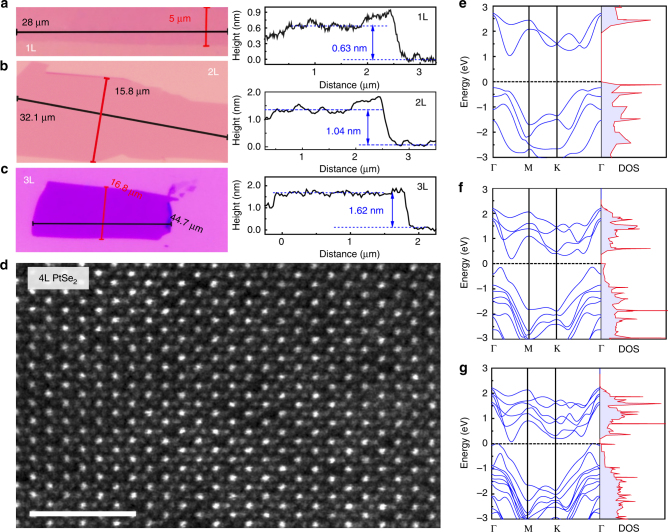
Characterization of atomically thin PtSe_2_ and the bandgap evolution obtained by first-principles calculations. **a**–**c** Optical microscope images and atomic force microscope (AFM) height profiles of monolayer, bilayer, and trilayer PtSe_2_. **d** HRTEM image of the atomic layered of PtSe_2_. The scale bar is 2 nm. **e**–**g** Density-of-states (DOS) of defect-free monolayer (**e**), bilayer (**f**), and trilayer PtSe_2_ (**g**) by first-principles calculations

PtSe_2_ atomic layers are mechanically exfoliated from the bulk crystals by the Scotch tape method, and transferred onto a silicon wafer with a 285-nm thermal oxidation layer. The optical microscope images of the exploited monolayer, bilayer, and trilayer PtSe_2_ on the Si/SiO_2_ wafer are shown in Fig. 3a–c. The thickness of the samples can be determined by the AFM height profiles, measured from the red lines crossing the flakes. The thickness of the monolayer PtSe_2_ is about 0.63 nm, as shown in Fig. 3a, where a slight dip in the boundaries is caused by the absorbed molecules and/or residues. As shown in Fig. 3b, c, the thicknesses of the bilayer and the trilayer PtSe_2_ are measured to be about 1.04 nm and 1.62 nm, respectively, which almost increase linearly from monolayer to trilayer. The layer-dependent properties can also be characterized by Raman spectroscopy, similar to other 2D material characterizations^18^. The synthesized bulk PtSe_2_ and atomic layers show two main Raman peaks near 200 cm^−1^ and 300 cm^−1^, as shown in Fig. 1c and Supplementary Figs. 3-4, which were defined as *E*_g_ mode and *A*_lg_ mode vibration, respectively^17^. On the other hand, the interlayer shear modes of atomic-layered PtSe_2_ in the ultra-low-frequency Raman spectrum, as shown in Supplementary Fig. 5, clearly indicate the layered structure of PtSe_2_ flakes. And it could be used to accurately determine the layer number of the few-layer PtSe_2_, as shown in Supplementary Fig. 6. In addition, it is also clear, as shown in the HRTEM image in Fig. 3d, that there are a considerable number of Se vacancies randomly distributed in the crystal lattice. These defects play an important role in the bandgap tuning and light absorption in the mid-IR regime of the PtSe_2_ atomic layers, and will be discussed in detail later.

To understand the layer-dependent band structure and bandgap evaluation of PtSe_2_, first-principles calculations are performed. It is shown that the monolayer and bilayer PtSe_2_ are semiconductors with indirect bandgap of ~1.2 and ~0.3 eV, respectively (see Fig. 3e–g), which are consistent with the earlier reports^16,19^. It becomes metallic without a bandgap for thicker PtSe_2_ layers. The slight mismatch in our calculations might be caused by the scanning resolution limit of the Vienna Ab Initio Simulation Package projector for such a narrow bandgap semiconductor, as shown in Supplementary Figs. 1 and 2. The narrow bandgap of the bilayer PtSe_2_ makes it suitable for light absorption in the mid-infrared (*λ* > 4 μm) region, filling up the gap between graphene and commonly explored BP and TMDCs. Thus, bilayer PtSe_2_ is an appropriate 2D semiconducting candidate with interband transition for mid-infrared photonic and optoelectronic applications.

### Optoelectronic properties of monolayer and bilayer PtSe2 FET

To demonstrate the optoelectronic properties of the atomically thin PtSe_2_ layers, we investigated mid-infrared photodetection with PtSe_2_ FET as a demonstrative example to show its practical applications. The electrical contacts made of Ti/Au (20 nm/80 nm) layers are deposited by the e-beam evaporation, after standard photolithography procedures. Heavily doped Si substrate is used as the back gate. The *I*_D_ - *V*_D_ and *I*_D_ - *V*_G_ curves are measured by the Agilent probe station, as shown in Supplementary Figs. 9−11. All the measurements are conducted in the dark under room temperature. One striking observation is the layer-dependent metallic to semiconducting phase transition: trilayer PtSe_2_ was metallic, while bilayer and monolayer PtSe_2_ were semiconducting, which is consistent with the theoretical predictions and experimental results^17,19^. It also depicts that both bilayer and monolayer PtSe_2_ exhibit classical n-type semiconducting behaviors. Additionally, the carrier mobilities of bilayer and monolayer PtSe_2_ can be calculated to be 8.6 and 1.7 cm^2^ V^−1^ s^−1^, respectively, which are on par with previous reports about InSe^20,21^, In_2_Se_3_^22,23^, and so on. The decrease of the mobility from bilayer PtSe_2_ FET to monolayer PtSe_2_ FET might be attributed to the surrounding conditions or the charge transfer from neighboring adsorbates and the substrates^24^.

**Fig. 4:**
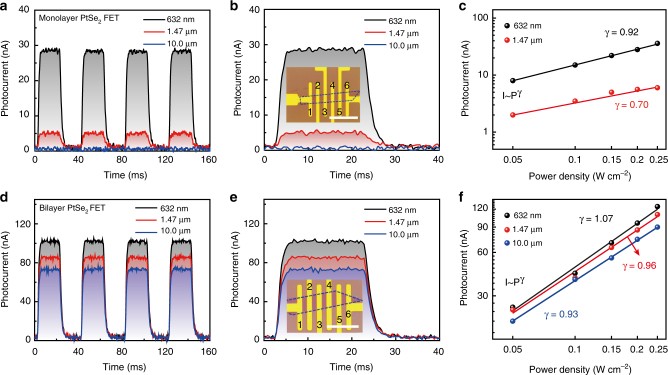
Optoelectronic properties of the monolayer and the bilayer PtSe_2_. **a**, **b** Time-resolved photoresponse of the monolayer PtSe_2_ field effect transistor (FET) devices for a bias voltage of 0.1 V and zero gate voltage under laser illumination with a wavelength of 632 nm, 1.47 µm and 10 µm, respectively. **c** Power dependence of the monolayer PtSe_2_ FET devices under laser illumination recorded at *V*_D_ = 0.1 V. **d**, **e** Time-resolved photoresponse of the bilayer PtSe2 FET device. The laser illuminations in **a**, **b**, **d** and **e** are kept in a constant of 0.2 W cm^−2^ for three different lasers. **f** Power dependence of the bilayer PtSe_2_ FET devices under laser illumination recorded at *V*_D_ = 0.1 V. The insert figures in **b** and **e** are the microscopic image of the monolayer and bilayer PtSe_2_ FET, respectively, measured in the experiment. The blue envelope area is the PtSe_2_ sample. The scale bar is 20 µm

We then discuss the photodetection performance of monolayer PtSe_2_ FET. The measurement setup is shown in Supplementary Note 4, and the power concentration of the laser illumination is kept in constant for time-resolved measurement, as shown in Fig. 4a, b. The time-resolved photoresponse-measurement results of monolayer PtSe_2_ are shown in Fig. 4a,b and Supplementary Table 1. The photocurrent is defined as *I*_ph_ = *I*_illum_ − *I*_dark_, where *I*_dark_ is the dark current and measured to be 28.5 nA and 5.5 nA for 632 nm and 1.47 µm illumination, respectively. The photodetectors based on monolayer PtSe_2_ show photoresponses of 0.9 and 0.15 A W^−1^ for 632 nm and 1.47 µm illumination, respectively. However, negligible photoresponse under a mid-infrared laser illumination (*λ* = 10 µm) is observed because the bandgap of monolayer PtSe_2_ (*E*_g_ ~ 1.2 eV) far exceeds the photon energy of the incident laser.

On the other hand, bilayer PtSe_2_ FET with controlled defect engineering exhibits a much higher photoresponse, compared with the monolayer PtSe_2_ FET, as shown in Fig. 4d, e. For instance, the photoresponsivities (*R*_res_) in the visible and near-infrared regions are about 6.25 A W^−1^ and 5.5 A W^−1^, respectively. Importantly, we also observe a strong photoresponse in the mid-infrared region (*λ* = 10 µm), as shown in Fig. 4d, and a photoresponsivity of ~4.5 A W^−1^ as shown in Supplementary Table 2, which corresponds to an internal gain of 17.5 (see supplementary materials). The photoresponse of the bilayer PtSe_2_ is about three orders higher than common graphene photodetectors and on par with the commercial MCT and QWIP detectors. Furthermore, the rise time and decay time can be fitted by the following equation^25^: *I*_rise_ = *I*_0_ − *A* exp(−(*t* − *t*_1_)/*τ*_1_) and *I*_decay_ = *I*_0_ + *B* exp(−(*t* − *t*_2_)/*τ*_2_), where *τ* is the time constant and *t* is the time when the laser is switched on or off. *A* is the scaling constant. The fitted characteristic photoresponse time coefficients, *τ*_1_ and *τ*_2_ are 1.2 ± 0.1 ms for rise time and 1.2 ± 0.1 ms for fall time, respectively, which is better than those of other types of 2D semiconducting photodetectors, such as the reported MoS_2_ photodetectors^24,26,27^ and BP photodetectors^28–32^. Besides, the rise time and fall time can also be calculated by the time period between 10% and 90% of the current in the rising and falling curves, as shown in Fig. 4b, e. The rise time (*τ*_1_ = 1.1 ms) and fall time (*τ*_2_ = 1.2 ms) are consistent with the former-fitted values. Last but not least, it is important to mention that the detectivity (D^*^) of PtSe_2_-based mid-infrared photodetector, which is an essential figure of merit for the detector, is limited by the 1/*f* noise other than the shot noise in our operation conditions. We measure the 1/*f* noise of the device in the frequency range from 1 Hz to 10 kHz, as shown in Supplementary Fig. 12. It is clearly indicated that the low-frequency 1/*f* noise prevails in our photodetectors, similar to other 2D systems^33^.The detectivity can be calculated based on the current noise spectrum, as shown in Supplementary Fig. 13, and noise the equivalent power (NEP, supplementary materials) in the current noise spectra by ${\it{D}} = \sqrt {{\it{AB}}} {\it{/NEP}}$, where *A* is the area of the detector and *B* is the bandwidth. The detectivity is calculated to be ~7×10^8^ Jones (cm Hz^1/2^ W^−1^) under 10 μm quantum cascade laser (QCL) illumination, which is at the same level as that of commercial mid-infrared photodetector, in this wavelength range^2^.

In addition, the power-dependent photocurrent can be expressed by the power law, *I*_PC_ = *CP*^*γ*^ (*C* is a constant and *P* is the illumination power), as shown in Fig. 4c, f. For the laser at the operating wavelengths of 632 nm and 1.47 µm, *γ* is 0.92 and 0.7 for monolayer PtSe_2_ FET, respectively, indicating that the recombination kinetics of the photocarriers involves both traps/defects states and photogenerated carrier interactions^34^. In comparison, the value of *γ* is 1.07, 0.96, and 0.93 for 632 nm, 1.47 µm, and 10 µm for bilayer PtSe_2_ FET, respectively. The relatively higher value for bilayer PtSe_2_ FET might be ascribed to mid-gap states and substrate effects, compared to the monolayer devices^24^. Further investigation is needed to interpret the complex-carrier recombination and scattering processes of the photogenerated electron/hole pairs. Furthermore, the increase of the photocurrent with the incident laser power, as shown in Fig. 4c, f, together with the laser wavelength-dependent photoresponse, as shown in Supplementary Fig. 14, indicates increase in the number of photogenerated carriers with an increase in the incident laser power^26^.

It is also noted that, the response speed of the bilayer PtSe_2_ FET is quite fast at the millisecond level, which surpasses the previously reported results of graphene nanostructured photodetectors^35,36^ and 2D semiconducting photodetectors^26^. In addition, the mid-infrared photodetection performance can be further improved by dielectric engineering and surface engineering strategies^37^. For example, the mobility of the PtSe_2_ FET could be enhanced in the high-dielectric environment due to the reduction of Coulomb interactions among the carriers in the channel. Furthermore, the slow response speed might be originated from the trapping states in the active channel, which can be optimized by surface-engineering methods, such as chemical treatment and trapping molecular/film decorations^36,37^.

## Discussion

**Fig. 5:**
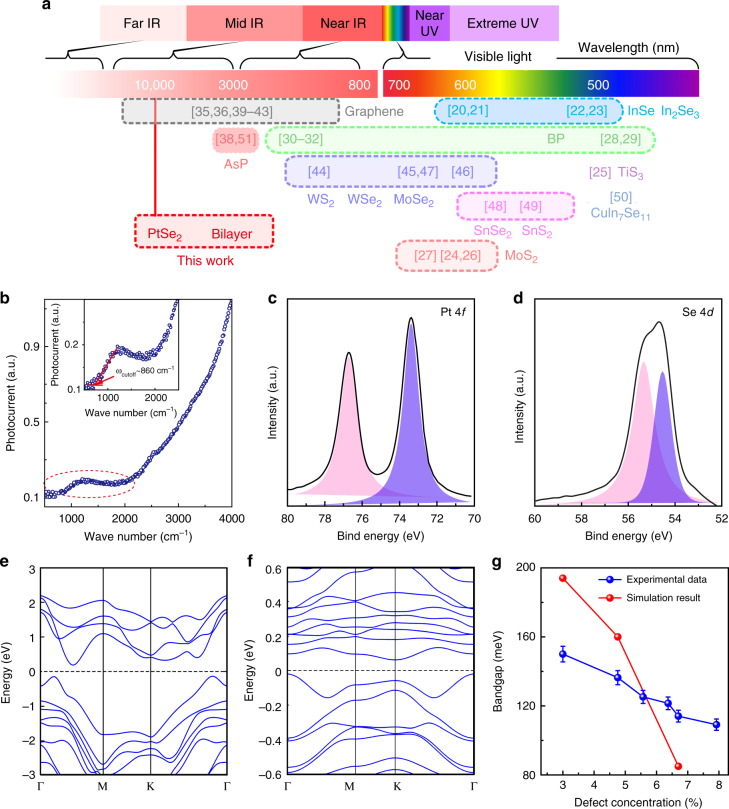
Narrow bandgap bilayer PtSe_2_ via defect engineering. **a** Comparison of photodetectors operated at different wavelengths, based on two-dimensional materials, such as graphene^35,36,39–43^, MoS_2_^24,26,27^, black phosphorene^28–32^, WS_2_^44,45^, WSe_2_^46^, MoSe_2_^47^, SnSe_2_^48^, SnS_2_^49^, InSe^20,21^, In_2_Se_3_^22,23^, TiS_3_^25^, CuIn_7_Se_11_^50^, and black AsP^38,51^. The mid-infrared range is rarely explored besides graphene. **b** Photoresponse spectrum of the photodetector based on the Ar plasma treatment bilayer PtSe_2_ FET (20 s), measured by FTIR internal source. **c**, **d** X-ray photoelectron spectroscopy (XPS) spectrum of Pt 4*f* (**c**) and Se 3*d* (**d**) core level peaks of PtSe_2_ with Se vacancies. **e**, **f** Density of states (DOS) of bilayer PtSe_2_ with 3% (**e**) and 6.8% (**f**) Se vacancy concentrations by first-principles calculations. **g** The experimental (blue) and theoretical (red) bandgap evolution of bilayer PtSe_2_ with different Se vacancy concentrations

As shown in Fig. 5a, mid-infrared detection with 2D materials has been a challenging task due to the lack of suitable candidates, besides graphene sheet and graphene nanostructures^35^. In this work, we report bilayer PtSe_2_ with designed defect engineering, for the first time, as a broadband mid-infrared photodetector operated at room temperature, as shown in Fig. 5b and Supplementary Table 2. The responsivity and the response speed also far exceeds those obtained in the recently discovered black arsenic phosphorus in the 3–5 µm range^38^. The broadband photoresponse of bilayer PtSe_2_ FET is originated from the defect-induced bandgap decrease. We now turn to the discussion of the additional characterizations and the reasons that address potential explanations of these findings. The quality and stoichiometric ratio between Pt to Se are further monitored by XPS, as shown in Fig. 5c, d and Supplementary Fig. 7. From the calculations, and based on the XPS spectra, we obtained the atomic ratio of Se/Pt of 64/36, which agrees well with the EDS spectrum and HRTEM images, as shown in Fig. 2 (see supplementary materials). In order to examine the role of defects in the electronic properties of bilayer PtSe_2_, we developed a technique to control the defect concentration via argon (Ar) plasma treatment in this home-made system.

After Ar plasma treatment with controlled dosages, the defect concentration and evolution of the bandgap of bilayer PtSe_2_ with the increase of defect concentration are characterized by the XPS spectra and FTIR absorption measurements, as shown in Supplementary Fig. 8, where the absorption cut-off wavelength dramatically shifts from 1210 cm^−1^ to 870 cm^−1^ (corresponding to photon energy from 150 meV to 110 meV) with the defect concentration increase from 3% to 7.9%. Consequently, the bandgap evolution with introducing Se-vacancy through controlled Ar plasma treatment is also verified by the photoresponses (10 µm laser illumination) of the bilayer PtSe_2_ samples, as shown in Supplementary Fig. 15, where the photoresponse increases dynamically with the increase in the defect concentration. Furthermore, we investigate the bandgap evolution of bilayer PtSe_2_ with different concentrations of Se vacancies, using first-principles calculations; the results are shown in Fig. 5e, f. The decrease of the bandgap of bilayer PtSe_2_ with the increase of the defect concentration can be explained as below. The valence band maximum in bilayer PtSe_2_ is mainly contributed by 5*d* orbitals of Pt atoms that contains rich *d*-electrons. As a result, the interaction of the *d* band of Pt atom and the *p*_*z*_ band of Se atom thermodynamically favors to form *sp*^*3*^*d*^*2*^ hybridization (1*T* phase), in which, less *d* orbitals are involved and the bond between Pt and Se are weaker than those of group-5 or group-6 TMDCs, such as MoS_2_, WS_2_ with *spd*^*4*^ hybridization in 2*H* phase. During the synthesis procedure and Ar plasma treatment, high temperature is required to exceed the melting temperature of Pt, which breaks the Pt−Se bond and causes Se vacancies. From the DOS of bilayer PtSe_2_ with defects, it is shown that the mid-gap states created by Se vacancy are located in the vicinity of the Fermi level and thus decrease the bandgap of the bilayer PtSe_2_. The fact that only the samples with controlled Ar plasma treatment processes a bandgap smaller than the excitation photon energy and exhibit distinct photoresponse validates our theoretical predictions of the bandgap evolution of bilayer PtSe_2_ via defect engineering. The presented results indicate the importance of Se vacancy over the bandgap of PtSe_2_ atomic layers, suggesting that defect engineering could be an effective strategy to control the bandgap and the electronic structure of PtSe_2_ atomic layers.

In summary, we successfully synthesize a narrow bandgap 2D PtSe_2_ crystal and obtain large area PtSe_2_ with atomic layers. The trilayer and thicker PtSe_2_ are semi-metallic, while bilayer and monolayer PtSe_2_ are *n*-type semiconductors with indirect bandgap. Significantly, it is experimentally proved that the bandgap can be further modulated through defect engineering (e.g., varying Se vacancy) to cover the mid-IR band continuously, which is important for mid-infrared photonics and optoelectronics. As a demonstrative example, we obtain the photodetection properties of the monolayer and the bilayer PtSe_2_ FET devices, and find that monolayer PtSe_2_ FET devices are suitable for visible and near-infrared photodetectors, and bilayer PtSe_2_ FET devices are suitable for broadband mid-infrared photodetectors. The broadband ranges from the visible to the mid-infrared along with high responsivity and fast-response speed. These results show that PtSe_2_ and other narrow bandgap TMCs are highly promising platforms for novel atomically thin optoelectronic applications, especially in the mid-infrared region, and they could be used to further diversify the family of 2D materials.

## Methods

### Experimental section

Synthesis of PtSe_2_ crystal flakes: The experiment setup is shown in Fig. 1c. The charges of platinum (powder, 99.9%, Sigma-Aldrich), selenium (powder, 99.9%, Sigma-Aldrich), red phosphorus (lump,99.9%, Sigma-Aldrich), and S (powder, 99.99%, Sigma-Aldrich) in the molar ratio of 1:2:1:3 with a total weight of 700 mg plus 35 mg I as the transport gas were sealed in an evacuated 20 cm long quartz tube under vacuum at 10^–6^ Torr, which was placed in a three-zone furnace. Firstly, the charge pre-reacted at 900 °C for 40 h. Secondly, at a lower temperrature of 700 °C for 5 days, the temperature of the reaction zone did not change with the growth zone to form a temperature gradient at which the growth of the single crystals take place, and finally, the furnace was naturally cooled down to room temperature in order to get good single crystals of PtSe_2_. Alternative ratios were tried, but the ratio of Pt:Se:P:S in 1:2:1:3 is optimal. In addition, the single crystals of PtSe_2_ cannot be obtained without P and S, which are necessary to obtain the high-quality single crystals and play a catalytic role in the growing process of PtSe_2_ single crystals.

### Material characterization

Raman data are collected at room temperature using 532 nm laser as the excitation source (WITec alpha 300 RAS Raman system). The sample size, thickness, morphologies, and atomic structures were conducted by optical microscopy (Olympus BX51), AFM, TEM, and high-resolution STEM.

### Electrical and optical characterization

The electrical characteristics are examined by a semiconductor analyzer (Agilent, B1500A). The photoresponsivity measurement is performed in a digital deep-level transient spectroscopy (BIORAD) system with different lasers to illuminate the whole device with the semiconductor diode (632 nm), fiber laser (1.47 µm, FC-W-1470), and quantum cascade laser (10 µm QCL, DAYLIGHT TLS-41105). The lasers are focused by a parabolic mirror followed by a mechanical chopper with a frequency controller. The noise spectra is acquired by a spectrum analyzer (Keysight M9018A, with a measuring bandwidth of 10 kHz) with source-drain bias supplied by Agilent 1500A at ambient conditions.

### Data availability

The data that support the plots with this paper and other findings of this study are available from the corresponding author upon reasonable request.

## Electronic supplementary material

Supplementary Information
