# Supplementary material for: Atomically thin noble metal dichalcogenide: a broadband mid-infrared semiconductor
Source: Nat Commun. 2018 Apr 18;9:1545. doi: 10.1038/s41467-018-03935-0 (PMC5906448; doi:10.1038/s41467-018-03935-0)
Supplement: Supplementary file 1 — Supplementary Information [file 41467_2018_3935_MOESM1_ESM.pdf]

Atomically-thin Noble Metal Dichalcogenide: A Broadband Mid-infrared Semiconductor

Yu. et al.

Supplementary Note 1

Computational details. Electronic structures are calculated within the density functional theory (DFT)[1] framework with the projector augmented wave (PAW) basis using the VASP (Vienna Ab Initio Simulation Package) codes[2-4]. The Perdew–Burke–Ernzerhof-type generalized gradient approximation (GGA) is used to describe the exchange-correlation energy[1]. The spin-orbit coupling (SOC) is included self-consistently to include the relativistic effects[5, 6]. To model different atomic layers structure of $PtSe_2$, we employed slab model with a vacuum of 15Å to avoid interaction between the periodically repeated slabs. A plane wave cutoff energy of 500 eV is used. The Brillouin zone sampling is done by using 12×12×12 and 12×12×1 Gamma-centered *k*-meshes for bulk and slabs, respectively. The defect calculations are performed using the supercell geometry of the corresponding unit cell. The total energies in the calculations are converged to 1×10^{-6} eV and the atomic positions are relaxed until the residual forces on each atom are less than 1×10^{-3} eV/Å.

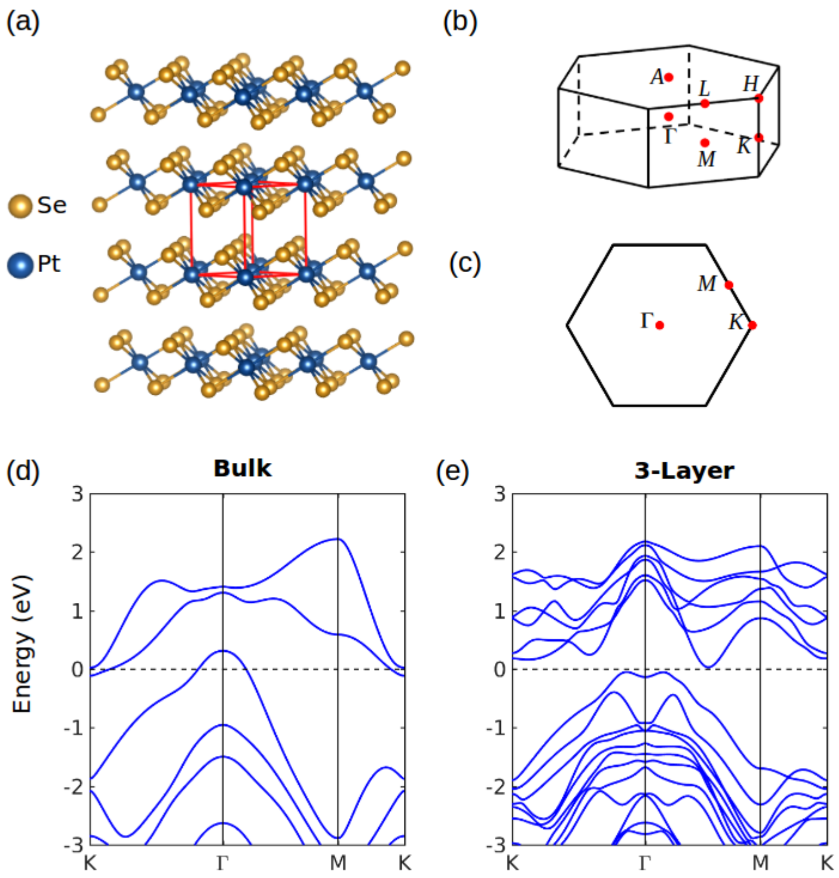

Supplementary Figure.1. (a) Crystal structure of multilayer stack (4 layers) of $PtSe_2$. Red box indicates the bulk hexagonal unit cell. Brillouin zone of (b) 3D bulk and (c) 2D thin films

of $PtSe_2$ in which high symmetry points are marked. (d) Band structure of bulk $PtSe_2$. (e) Band structure of trilayer $PtSe_2$.

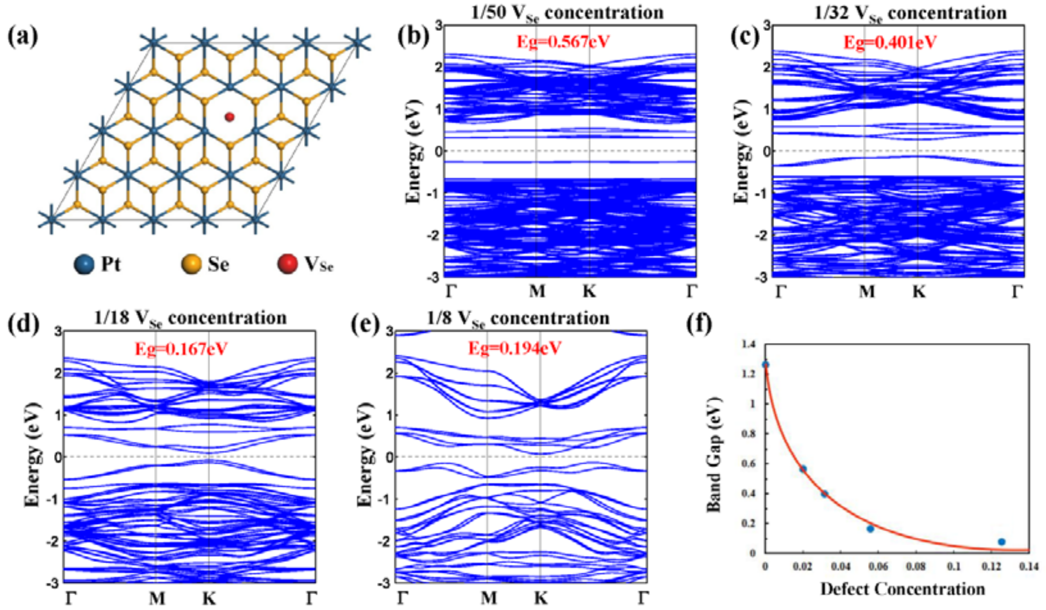

Supplementary Figure.2. Defect engineering of monolayer $PtSe_2$. (a) Illustration of 4×4×1 supercell crystal structure of monolayer $PtSe_2$ with Se vacancy. Red ball indicates the Se vacancy. (b)- (e) Band structures of monolayer $PtSe_2$ with different defect concentrations, 1/50 (b), 1/32 (c), 1/18 (d) and 1/8 (e). (f) Bandgap evolution of $PtSe_2$ monolayer with defect concentrations. Blue dots are obtained from calculations, and a line is added to guide the eyes.

Supplementary Note 2

Raman spectrum of $PtSe_2$ atomic layers.

The layer-dependent properties can also be characterized by Raman spectroscopy similar to other two-dimensional materials[7]. The synthesized bulk $PtSe_2$ and atomic layers show two main Raman peaks near 200 cm^{-1} and 300 cm^{-1} as shown in Figs. S3 and S4, which are defined as E_g mode and A_{1g} mode vibration, respectively[8]. It was also obvious that the E_g and A_{1g} vibration mode change drastically with the decrease of the layers of $PtSe_2$. We believe the variation of the peak intensity ratio can be employed as a signature to distinguish the number of $PtSe_2$ layers, and the variation modes of the Raman peaks needs to be explored theoretically in the next section. However, we noticed that the fingerprint of the Raman spectrum in Fig. S3 is not very sensitive to the layer variations.

Alternatively, ultralow-frequency (ULF) Raman spectroscopy has been widely used for the characterization of two-dimensional materials, which is extremely sensitive to the number of layer and the layer configurations[9, 10]. As clearly shown in Figs. S5 and S6, the ultralow-frequency mode around 10 cm^{-1} to 40 cm^{-1} was observed for monolayer, bilayer and trilayer $PtSe_2$ flakes. The ULF modes strongly depended on the layer number (N) and absent in monolayer because they were originated from the interlayer shearing[11]. With decreasing N, the shear modes frequency decreased rapidly due to the reduced effective interlayer spring constant. The shear mode frequency (ω_s) could be quantitatively analyzed as $\omega_S = \omega_0 \cos(\pi / 2N)$, where ω_0 is the bulk shear mode frequency that is absent in our measurement. By fitting the ULF Raman spectroscopy, we obtained $\omega_0 = 32$ cm^{-1} for bulk $PtSe_2$ materials.

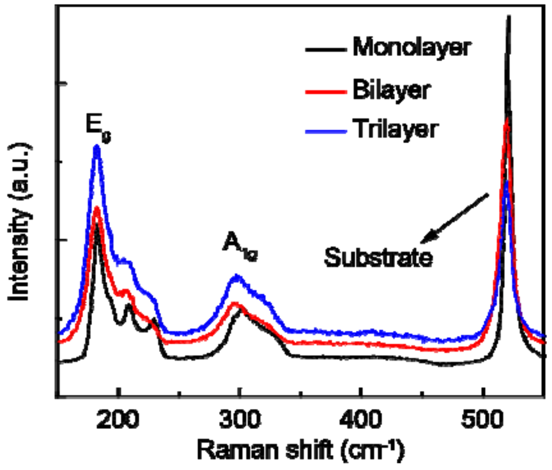

Supplementary Figure 3. Raman spectroscopy of monolayer, bilayer and trilayer $PtSe_2$ exfoliated by scotch tape on Si/SiO_2 wafer.

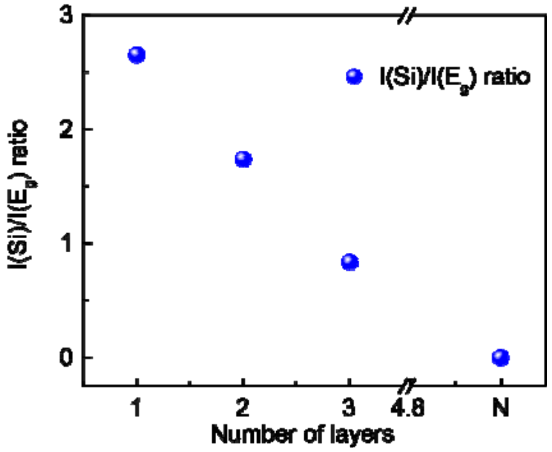

Supplementary Figure 4. Comparison of the intensity of Raman peaks between Si (520 cm^{-1}) and E_g peak of $PtSe_2$.

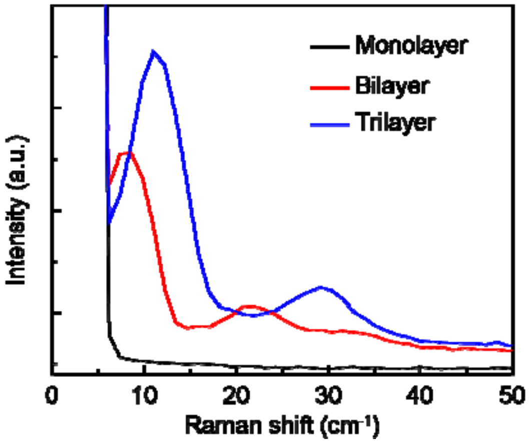

Supplementary Figure 5. Ultralow frequency Raman spectroscopy of monolayer, bilayer and trilayer $PtSe_2$. As discussed in the manuscript, ULF Raman spectrum is layer-sensitive because ULF is attributed to the interlayer coupling, thus it can be employed as a probe to characterize the layers of $PtSe_2$ flakes.

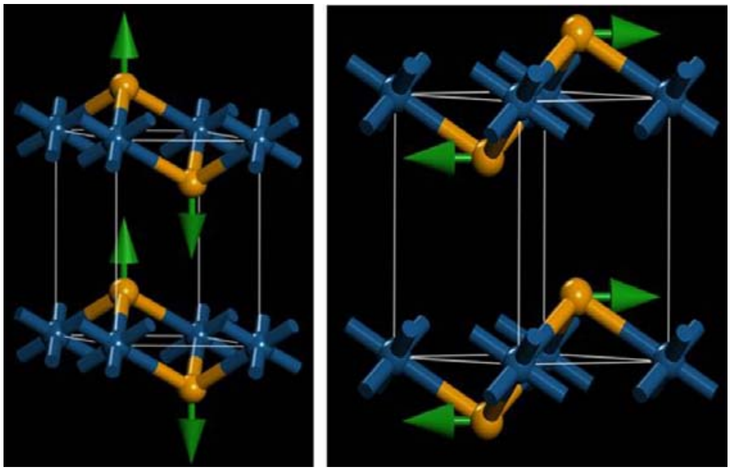

Supplementary Figure 6. Simulation model for the Raman spectroscopy of atomic layered $PtSe_2$. The left and right figure indicates the A_{1g} mode and E_g mode vibrations of $PtSe_2$.

Supplementary Note 3

Optical absorption and bandgap evaluation of bilayer $PtSe_2$

The defects in $PtSe_2$ atomic layers can be controlled by two methods: temperature control during the synthesis process and Ar plasma treatment. The Oxford Plasma Pro Cobra 100 Deep RIE was employed to generate low-dose Ar plasma to generate Se defects in atomically thin $PtSe_2$ layers, in which the defect concentration is dependent on the treatment time. The defect concentration can be characterized by the XPS as shown in Fig. S7. The atomic ratio can be calculated by the semi-quantitative analysis through measuring the peak areas of Se and Pt core lines (I) and applying the appropriate atomic sensitivity factors of both elements (S) which is known as the relative sensitivity factors (RSF): $C_x = I_x S_x / \sum I_i S_i$, where C_x is the atomic fraction of element x in the sample. As a result, the defect density can be calculated accordingly.

As explained in the manuscript, bilayer $PtSe_2$ is suitable for mid-infrared optoelectronic applications. As a result, we systematically investigate the bandgap evaluation of bilayer $PtSe_2$ with different defects. The bilayer $PtSe_2$ samples are mechanically exfoliated and then transferred to thin KBr substrates. The pristine samples are synthesized by CVT method, which has intrinsic defects introduced by the reaction processes. To further control the defect density of the bilayer $PtSe_2$ samples, Ar plasma treatment are employed. The parameters such as RF power and arcing times are shown in the experimental details. The absorption of the bilayer $PtSe_2$ samples are measured by a micro-FTIR system (BRUKER VERTEX 70) and are indicated as below figure. The results show that the defect created by Ar plasma treatments plays a beneficial role in boosting the optical absorption in the infrared range. Furthermore, the absorption cut-off of each sample decreases gradually from ~1210 cm^{-1} (pristine sample) to 870 cm^{-1} ($PtSe_2$ sample treated by Ar plasma for 20 s) by increasing the plasma treatment time.

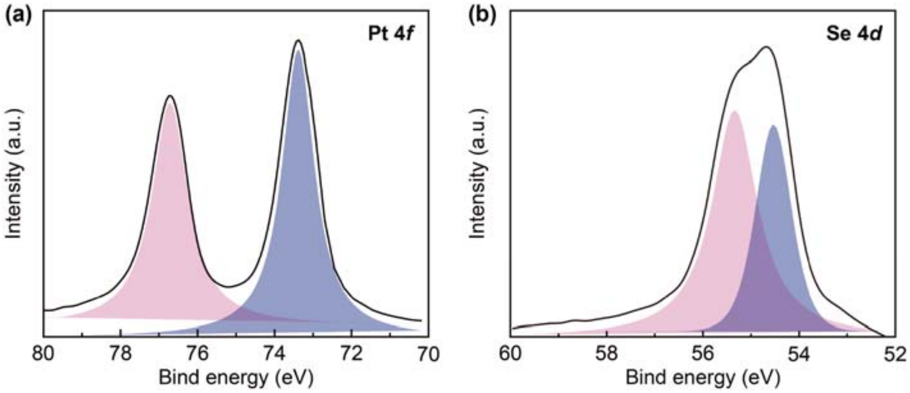

Supplementary Figure 7. XPS spectrum of the $PtSe_2$ samples with Ar plasma treatments.

It is clearly shown in the XPS spectrum that Se atom is insufficient in the fresh samples. The two dominant peaks in the Se *3d* spectrum (55.40 eV and 54.50 eV) exhibit the dominance of Se^{2-} peaks and full crystallization of $PtSe_2$, which are slightly higher than the bonding energies of Se^{2-} states and can be deciphered by the change in the chemical state of Se atoms in the presence of Se vacancies. Ar plasma treatment further decreased the Se atom ratios because the anions (chalcogen) are easier to dissociate from the crystal than cations. The defect concentration can be controlled by arcing period. The dependence of the Se defect concentration *vs* etching time is shown in Fig. S8. From the XPS spectrum, HRTEM and our DFT calculations, it is clearly shown that the Se vacancies can be well controlled by the fabrication and plasma treatment processes.

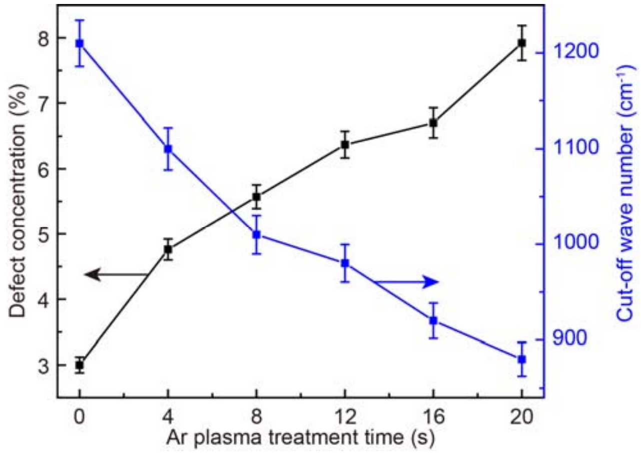

Supplementary Figure 8. The evolution of Se defect density and absorption cut-off wave numbers with increasing Ar plasma treatment steps. The steps are defined by the plasma arcing time (step 1= 4 s; step 2= 8 s; step 3= 12 s; step 4= 16 s; step 5= 20 s;).

Supplementary Note 4

Electrical measurements of atomic layered $PtSe_2$ based FET

Atomic-layer graphene flakes were mechanically exfoliated from the CVT sythnsized $PtSe_2$ single crystals using adhesive 3M-tape and deposited on a silicon wafer with a 285-nm thermalized SiO_2 layer. The location and quality of atomic $PtSe_2$ layers were identified by optical contrast using an optical microscope and Raman spectroscopy. Then, $PtSe_2$-based FETs with the heavily doped silicon substrate as a backgate electrode by standard photolithography and e-beam evaporation. The electrical characteristics were examined by a semiconductor analyzer (Agilent, B1500A). The mobility of the carriers can be calculated by $\mu = \frac{\mathrm{L}}{W \times (\varepsilon_0 \varepsilon_r / \mathrm{d})} \times \frac{dI_{ds}}{dV_G} \times \frac{1}{V_{ds}}$, where L, W and d denote the channel length, width and the thickness of SiO_2 layer (285 nm in our devices), respectively. V_{ds}, I_{ds} and V_b denote the source–drain bias, current, and bottom gate voltage in the linear region in the I_d–V_g curve. ε_0 and ε_r are the vacuum dielectric constant and the dielectric constant of SiO_2 (ε_r = 3.9), respectively. The photoresponsivity measurement was performed in a digital deep level transient spectroscopy (BIORAD) system with visible, near-infrared and mid-infrared lasers.

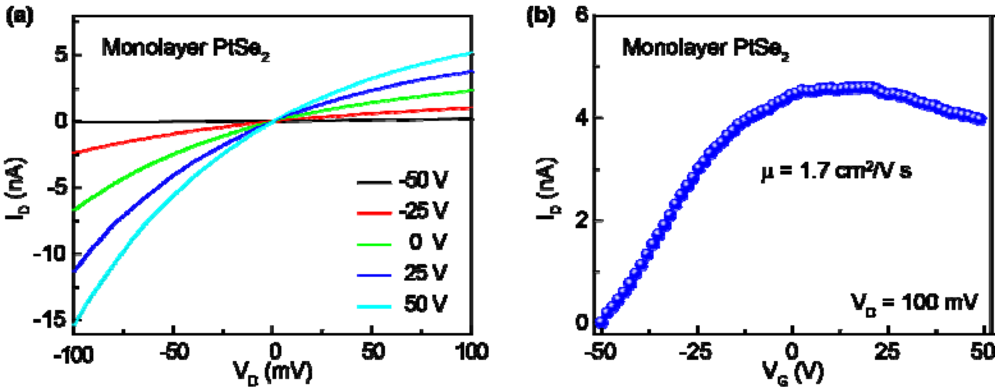

Supplementary Figure 9. I_d-V_d (a) and I_d-V_g (b) curve of monolayer $PtSe_2$ based FET. The calculated mobility of monolayer $PtSe_2$ FET is ~ 1.7 cm^2/V s.

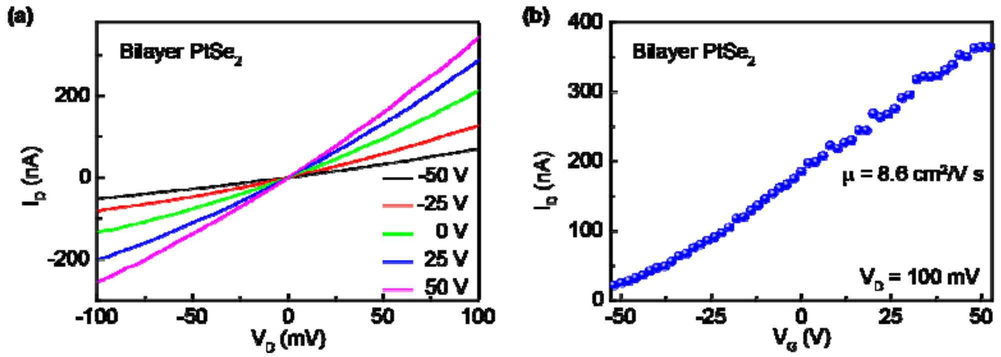

Supplementary Figure 10. I_d-V_d (a) and I_d-V_g (b) curve of bilayer $PtSe_2$ based FET. Bilayer $PtSe_2$ shows obvious semiconducting behavior with mobility of ~8.6 cm^2/V s.

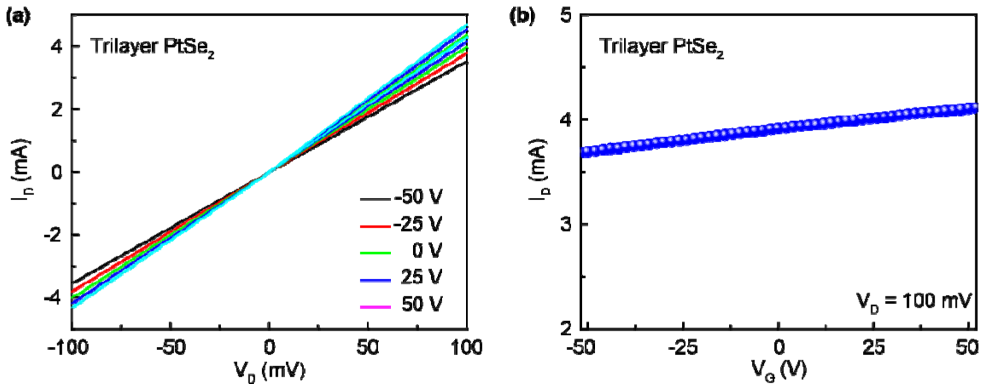

Supplementary Figure 11. I_d-V_d (a) and I_d-V_g (b) curve of trilayer $PtSe_2$ based FET. The electrical measurement indicates that trilayer $PtSe_2$ is metallic and shows negligible modulation by the gating voltage.

Supplementary Note 5

Noise spectra measurement

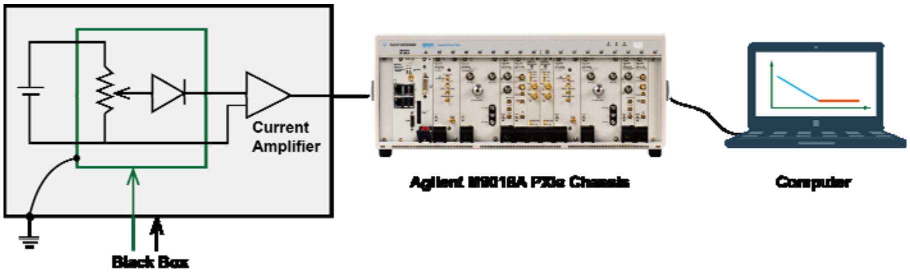

Supplementary Figure 12. Schematic setup of noise measurement.

To analyze the noise spectra and the detectivity of the photodetector, we measured the noise of the device at different drain-source voltage. The set-up is shown in Fig. S12. There are several types of noise in two-dimensional photodetectors calculated by the expression below[12, 13]. First of all, the low dark current that originated from the low bias voltage applied during the photodetector operation introduces a low shot noise (i_s) with the following expression:

$$i_s = \sqrt{2eI_d B} \quad (1)$$

where e is the electron charge, I_d is the dark current and B is the bandwidth. Based on the dark current of the device at 5 mV, the shot noise i_s is calculated to be 0.38 pA $Hz^{-1/2}$. Secondly, another important contribution of the noise of photodetector is the thermal noise (i_t) which can be calculated by the following expression:

$$i_t = \sqrt{\frac{4k_B TB}{R}} \quad (2)$$

where k_B is the Boltzmann constant, T is the operation temperature and R is the resistance of the detector. Based on the differential resistance at 5 mV as shown in Fig. S12, the thermal noise i_t can be calculated to be 0.12 pA $Hz^{-1/2}$. Therefore, the total white noise can be calculated to be 0.4 pA $Hz^{-1/2}$ by the expression below:

$$i_w = \sqrt{i_s^2 + i_t^2} \quad (3)$$

However, in the devices reported in this work, the operation speed is low and thus 1/f noise dominates at low frequencies. Basically, *1/f* noise originates from fluctuations of local electronic states induced by the disorder or defects. The current noise spectrum is shown in

Fig. S13 in the frequency range from 1Hz to 10 kHz. We calculate the *1/f* noise to be 17.3 pA $Hz^{-1/2}$. These results confirm that the *1/f* noise prevails at low frequencies for our device, which is similar to other two-dimensional transistors.[14-16]

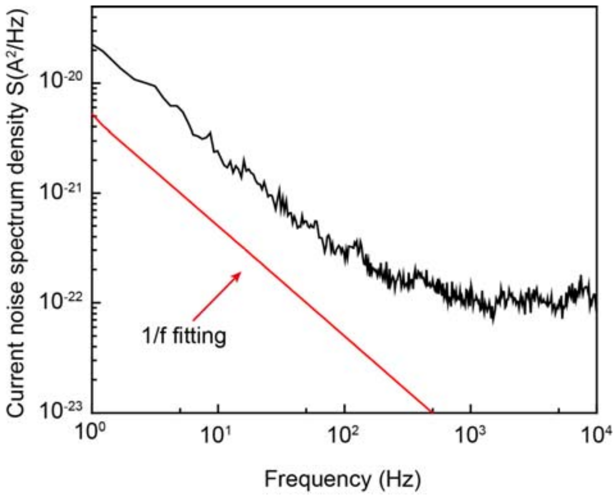

Supplementary Figure 13. The Current noise spectra at V_{ds}=5 mV of bilayer $PtSe_2$ FET device. The solid line is a reference for the 1/f noise trend and fitted by S~*1/f*.

Based on the current noise spectra and responsivity (R_{res}), noise equivalent power (NEP) and the detectivity (D^*) can be calculated according to the expressions below: [17-19]

$$R_{res} = \frac{I_{ph}}{P} \quad (4)$$

$$\mathrm{NEP} = \frac{i_n}{R_{res}} \quad (5)$$

$$D* = \frac{\sqrt{AB}}{NEP} \quad (6)$$

where I_{ph} is the photocurrent, P is the power illuminated on the active area, i_n is the measured noise, R_{res} is the responsivity and A is the active area of the device. The detectivity of our devices approaches 7×10^8 cm $Hz^{1/2}$ W^{-1}, which is much higher than that of commercial bolometers operated in this wavelength range. [19-21]

Supplementary Note 6

Photocurrent measurements of bilayer $PtSe_2$ FETs

Supplementary Table 1. Photoresponsivity measurements with different monolayer $PtSe_2$ samples divided by the finger-electrodes under the same conditions.

Parameters	Electrode 1-2	Electrode 2-3	Electrode 3-4	Electrode 4-5	Electrode 5-6
Sample area	2.3 μm ×6.7 μm =15.4 μm^2	2.7 μm ×6.7 μm =18.1 μm^2	2.7 μm ×6.7 μm = 18.1 μm^2	2.3 μm ×6.7 μm = 15.4 μm^2	2.3 μm ×6.7 μm = 15.4 μm^2
Responsivity (633 nm)	0.8 A/W	0.75 A/W	0.9 A/W	0.56 A/W	0.9 A/W
Responsivity (1.47 μm)	0.11 A/W	0.09 A/W	0.12 A/W	0.08 A/W	0.15 A/W

Supplementary Table 2. Photoresponsivity measurements with different bilayer $PtSe_2$ samples divided by the finger-electrodes under the same conditions.

Parameters	Electrode 1-2	Electrode 2-3	Electrode 3-4	Electrode 4-5	Electrode 5-6
Sample area	2.9 μm ×8.4 μm =24.3 μm^2	2.9 μm ×8 0 μm =23.2 μm^2	2.9 μm ×7.7 μm =22.3 μm^2	2.6 μm ×7.4 μm =19.2 μm^2	2.3 μm ×3.9 μm =8.9 μm^2
Responsivity (633 nm)	5.9 A/W	6.1 A/W	6.0 A/W	6.1 A/W	6.2 A/W
Responsivity (1.47 μm)	5.1 A/W	5.0 A/W	5.2 A/W	4.8 A/W	5.5 A/W
Responsivity (10 μm)	3.7 A/W	4.0 A/W	3.5 A/W	3.6 A/W	4.5 A/W

The internal gain can be calculated by the below equation[22, 23]:

$$\mathrm{Gain} = \frac{I_{ph} / e}{\left(\frac{S_{active}}{S_{laserspot}} \times P \times \alpha\right) / h\upsilon} \qquad (7)$$

where $\upsilon = \frac{c}{\lambda}$, c in the speed of light (3×10^8 m/s), λ is the wavelength of the incident laser (λ = 10 μm in our setup), S_{active} and $S_{laserspot}$ are the area of the sample and the laser spot, α is the light absorption and P is the power of the incident light calibrated by an existing IR photodetector.

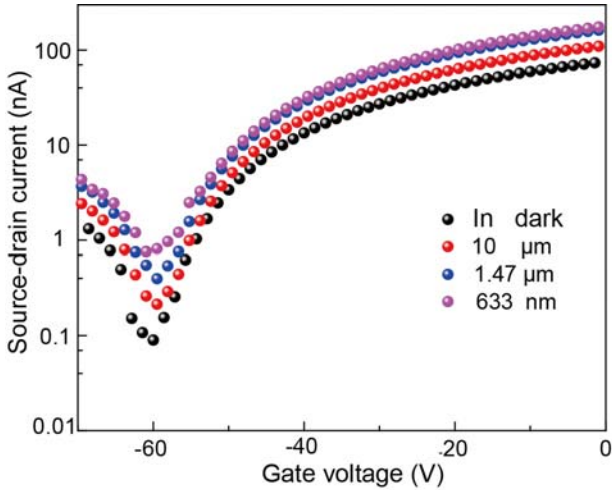

Supplementary Figure 14. Gate-dependence of the photocurrents with three different lasers illumination at 633 nm, 1.47 μm and 10 μm, respectively. The laser power is kept at 0.25 W/cm^2 constantly for all the three lasers.

We measured gate dependence photocurrent with 3 laser illuminations as shown in Figure S14, in which the devices present a weak conductive mid-gap states that is responsible for the charge transport in dark. The results indicate that the photocurrent generation is originated from the defect states mediated photoconductivity[21-23].

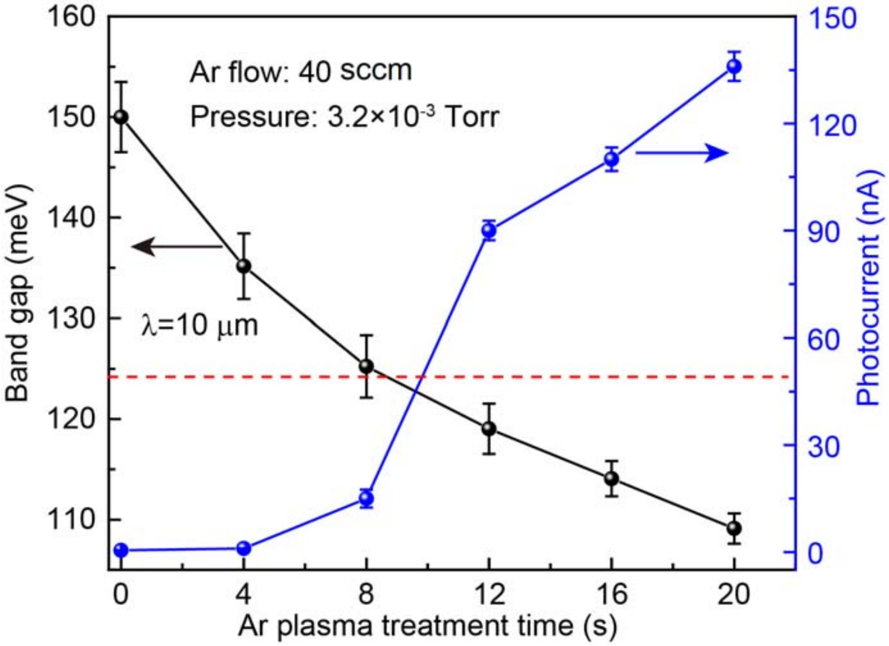

Supplementary Figure 15. Photocurrent and bandgap of bilayer $PtSe_2$ with deferent Ar plasma treatment time for increasing the defect density. The Ar plasma treatment parameters are indicated inside the figure: the Ar flow is 40 sccm and the vacuumof the chamber is kept at 3.2×10^{-3} Torr. The incident light is from a commercial quantum cascade laser (10 μm QCL,

DAYLIGHT TLS-41105). The source-drain bias for all the measurements are kept constantly at 0.1 V.

We fabricated the $PtSe_2$ FET samples with various defect concentration and measured the photoresponse with a quantum cascade laser (λ=10 μm). The excitation energy of the incident laser is ~ 124 meV. The photocurrents of all the samples are shown in Figure S15. From the bandgap evaluation of bilayer $PtSe_2$, only the ones by Ar treatment longer than 8s have the bandgap lower than the excitation energy, thus achieve detectable photocurrent. On the other hand, the photocurrent increases with a high defect concentration. These results clearly demonstrate the effect of defect concentration in bilayer $PtSe_2$ on the bandgap evaluation and the related optoelectronic properties.

References

1. Hohenberg, P.&Kohn, W. Inhomogeneous electron gas. *Phys. Rev.* **136**, B864-B871 (1964)
2. Kresse, G.&Furthmüller, J. Efficient iterative schemes for ab initio total-energy calculations using a plane-wave basis set. *Phys. Rev. B* **54**, 11169-11186 (1996)
3. Kresse, G.&Hafner, J. Ab initio molecular dynamics for liquid metals. *Phys. Rev. B* **47**, 558-561 (1993)
4. Kresse, G.&Joubert, D. From ultrasoft pseudopotentials to the projector augmented-wave method. *Phys. Rev. B* **59**, 1758-1775 (1999)
5. Perdew, J. P.; Burke, K.&Ernzerhof, M. Generalized gradient approximation made simple. *Phys. Rev. Lett.* **77**, 3865-3868 (1996)
6. Perdew, J. P., et al. Atoms, molecules, solids, and surfaces: Applications of the generalized gradient approximation for exchange and correlation. *Phys. Rev. B* **46**, 6671-6687 (1992)
7. Pisana, S., et al. Breakdown of the adiabatic born-oppenheimer approximation in graphene. *Nat. Mater.* **6**, 198-201 (2007)
8. Mingzhe, Y., et al. High quality atomically thin $PtSe_2$ films grown by molecular beam epitaxy. *2D Mater.* **4**, 045015 (2017)
9. Xi, X., et al. Strongly enhanced charge-density-wave order in monolayer $NbSe_2$. *Nat. Nanotechol.* **10**, 765-769 (2015)
10. Puretzky, A. A., et al. Low-frequency Raman fingerprints of two-dimensional metal dichalcogenide layer stacking configurations. *ACS Nano* **9**, 6333-6342 (2015)
11. Cong, C.&Yu, T. Enhanced ultra-low-frequency interlayer shear modes in folded graphene layers. *Nat. Commun.* **5**, 4709 (2014)
12. Choi, W., et al. High-detectivity multilayer MoS_2 phototransistors with spectral response from ultraviolet to infrared. *Adv. Mater.* **24**, 5832-5836 (2012)
13. Long, M., et al. Room temperature high-detectivity mid-infrared photodetectors based on black arsenic phosphorus. *Sci .Adv.* **3**, e1700589 (2017)
14. Zahid Hossain, M.; Rumyantsev, S.; Shur, M. S.&Balandin, A. A. Reduction of 1/f noise in graphene after electron-beam irradiation. *Appl. Phys. Lett.* **102**, 153512 (2013)
15. Liu, G.; Rumyantsev, S.; Shur, M. S.&Balandin, A. A. Origin of *1/f* noise in graphene multilayers: Surface vs. Volume. *Appl. Phys. Lett.* **102**, 093111 (2013)

16. Balandin, A. A. Low-frequency *1/f* noise in graphene devices. *Nat Nano* **8**, 549-555 (2013)
17. Dou, L., et al. Solution-processed hybrid perovskite photodetectors with high detectivity. *Nat. Commun.* **5**, 5404 (2014)
18. Adinolfi, V.&Sargent, E. H. Photovoltage field-effect transistors. *Nature* **542**, 324-327 (2017)
19. Rogalski, A. HgCdTe infrared detector material: History, status and outlook. *Rep. Prog. Phys.* **68**, 2267 (2005)
20. Haddadi, A., et al. $InAs/InAs_{1-x}Sb_x$ type-II superlattices for high performance long wavelength infrared detection. *Appl. Phys. Lett.* **105**, 121104 (2014)
21. Rogalski, A. History of infrared detectors. *Opto-Electron. Rev.* **20**, 279-308 (2012)
22. Koppens, F. H. L., et al. Photodetectors based on graphene, other two-dimensional materials and hybrid systems. *Nat. Nanotechnol.* **9**, 780-793 (2014)
23. Mak, K. F.&Shan, J. Photonics and optoelectronics of 2D semiconductor transition metal dichalcogenides. *Nat. Photon.* **10**, 216-226 (2016)
